# Non-contrast transcatheter aortic valve implantation for patients with aortic stenosis and chronic kidney disease: a pilot study

**DOI:** 10.3389/fcvm.2023.1175600

**Published:** 2023-06-14

**Authors:** Antônio Fernando Diniz Freire, Pedro Felipe Gomes Nicz, Henrique Barbosa Ribeiro, Filippe Barcellos Filippini, Tarso Duenas Accorsi, Gabriela Liberato, Cesar Higa Nomura, Renata de Sa Cassar, Marcelo Luiz Campos Vieira, Wilson Mathias, Pablo Maria Alberto Pomerantzeff, Flavio Tarasoutchi, Alexandre Abizaid, Roberto Kalil Filho, Fábio Sândoli de Brito

**Affiliations:** Department of Interventional Cardiology, Heart Institute of University of São Paulo (InCor), São Paulo, Brazil

**Keywords:** transcatheter aortic valve replacement, aortic stenosis, renal failure, zero contrast, renal insufficiency

## Abstract

**Background:**

Acute kidney injury (AKI) is frequently observed after transcatheter aortic valve implantation (TAVI). Of note, it is associated with a threefold increase in all-cause and cardiac death. We propose a new non-contrast strategy for evaluating and performing the TAVI procedure that can be especially valuable for patients with aortic stenosis (AS) and chronic kidney disease (CKD) to prevent AKI.

**Methods:**

Patients with severe symptomatic AS and CKD stage ≥3a were evaluated for TAVI using four non-contrast imaging modalities for procedural planning: transesophageal echocardiogram (TEE), cardiac magnetic resonance, multidetector computed tomography (MDCT), and aortoiliac CO_2_ angiography. Patients underwent transfemoral (TF) TAVI using the self-expandable Evolut R/Pro, and the procedures were guided by fluoroscopy and TEE. Contrast MDCT and contrast injection at certain checkpoints during the procedure were used in a blinded fashion to guarantee patient safety.

**Results:**

A total of 25 patients underwent TF-TAVI with the zero-contrast technique. The mean age was 79.9 ± 6.1 years, 72% in NYHA class III/IV, with a mean STS-PROM of 3.0% ± 1.5%, and creatinine clearance of 49 ± 7 ml/min. The self-expandable Evolut R and Pro were implanted in 80% and 20% of patients, respectively. In 36% of the cases, the transcatheter heart valve (THV) chosen was one size larger than the one by contrast MDCT, but none of these cases presented adverse events. Device success and the combined safety endpoint (at 30 days) both achieved 92%. Pacemaker implantation was needed in 17%.

**Conclusion:**

This pilot study demonstrated that the zero-contrast technique for procedural planning and THV implantation was feasible and safe and might become the preferable strategy for a significant population of CKD patients undergoing TAVR. Future studies with a larger number of patients are still needed to confirm such interesting findings.

## Introduction

Acute kidney injury (AKI) is frequently observed after transcatheter aortic valve implantation (TAVI), with rates ranging from 3% to 50% and up to 4.5% requiring dialysis (1). Of note, it may also occur in the pre-TAVI work-up, since the vast majority of such patients undergo contrast multidetector computed tomography (MDCT) evaluation (2). The occurrence of AKI has been related to the volume of contrast media used, in addition to the presence of comorbidities, such as chronic kidney disease (CKD), which is commonly observed among TAVI candidates (∼70%) (3–7).

It is noteworthy that AKI after TAVI is associated with poorer clinical outcomes, including a stepwise increase in all-cause death and cardiovascular mortality, according to AKI severity (8, 9). Also, it may be related to prolonged hospital stay, yielding high financial costs to the healthcare system (8). These data suggest that all efforts should be made to identify patients at risk and adopt preventive measures for AKI, such as avoiding the use of contrast media in the preprocedural evaluation and during the transcatheter heart valve (THV) implantation, especially in such CKD patients (10, 11).

Nonetheless, no study has specifically evaluated a strategy for performing the TAVI procedure, including the pre-procedural work-up, using a fully non-contrast strategy. The current study aims to evaluate the safety and feasibility of such a “zero-contrast” strategy using the self-expandable Evolut R/Pro THV.

## Methods

### Study design and population

Evolut zero-contrast TAVI was an investigator-initiated, prospective, single-arm, pilot study, evaluating the feasibility and safety of a fully non-contrast approach to perform the preprocedural planning and the transfemoral (TF) TAVI procedure, using the self-expandable Evolut R/Pro THV, in CKD patients (*clinicalTrials.gov*, *NCT04581694*). Patients presenting with severe symptomatic aortic stenosis (AS) and CKD stage ≥3a (glomerular filtration rate <60 ml/min/1.73 m^2^) were randomly selected from a waiting list of the Brazilian public health system. After enrollment in the present investigation, four non-contrast imaging modalities were used to evaluate these patients: (1) non-contrast multidetector computed tomography (MDCT), (2) aortoiliac carbon dioxide (CO2) angiography, (3) non-contrast magnetic resonance imaging (MRI) by two methods, 3D whole-heart and cine SSFP, and (4) three-dimensional (3D) transesophageal echocardiography (TEE). The roles of each of these non-contrast imaging modalities in procedural planning were as follows. (1) The feasibility of the femoral approach and the entry site was determined by the non-contrast MDCT and by the aortoiliac CO_2_ angiography performed in the screening phase. (2) The size of the Evolut (EV) R/Pro THV was defined based on the sinus of Valsalva (SOV) mean diameter as assessed by the non-contrast MDCT (Supplementary Table S1). The largest THV that could be accommodated in the SOV was chosen, and, in borderline situations, annulus perimeter by 3D TEE and MRI was used for size definition. (3) The distribution and intensity of the aortic valve and left ventricular outflow tract (LVOT) calcification was assessed by non-contrast MDCT. (4) Left and right coronary ostium height was evaluated by non-contrast MDCT and 3D TEE. (5) The optimal deployment projection for the TAVI procedure was defined by non-contrast MDCT.

In the pilot phase, patients were excluded if they were considered not suitable for the TF approach or if they were at a very high risk of annulus rupture, severe paravalvular leak (PVL), or coronary occlusion. A complete list of inclusion and exclusion criteria is shown in Supplementary Table S2. The study protocol was approved by the ethics committee, and all patients provided written informed consent.

### Endpoints

The primary endpoint of this pilot study was the early safety combined endpoint (at 30 days) including all-cause mortality, major stroke, life-threatening bleeding, acute kidney injury (stage 2 or 3), coronary artery obstruction requiring intervention, major vascular complication, and valve-related dysfunction requiring repeat procedure. The two secondary endpoints were (1) accuracy of the THV size chosen by the TAVI operator based on the non-contrast imaging modalities as compared to the THV size chosen by the gold-standard evaluation by ECG-gated MDCT with contrast and (2) device success defined according to VARC-2 criteria as the absence of procedural mortality, correct positioning of a single THV, and intended performance of the THV: no mismatch, mean aortic gradient <20 mmHg or peak aortic velocity <3 m/s, and no moderate or severe aortic regurgitation (AR).

### Patient safety

**Figure 1:**
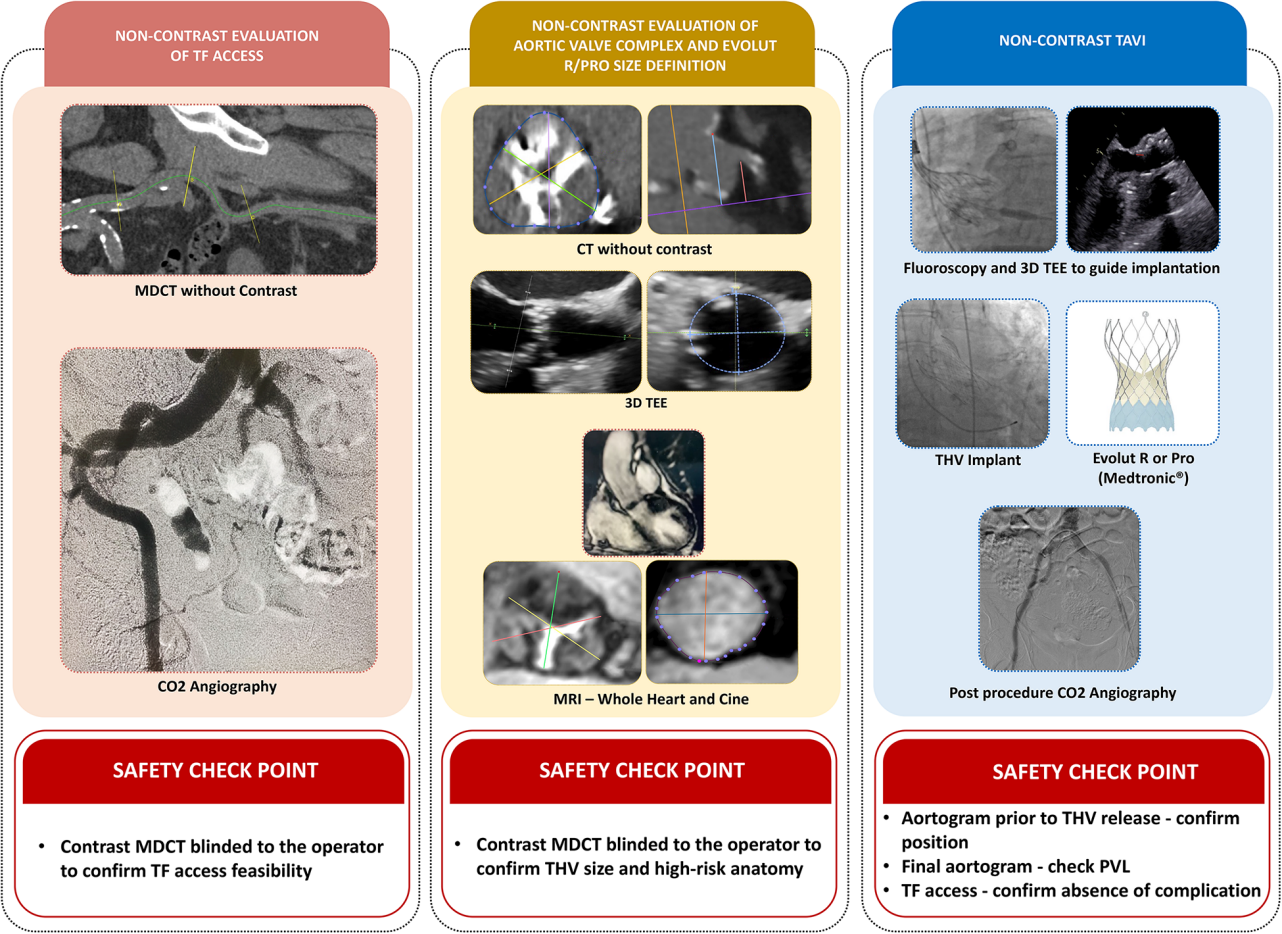
Central illustration. TAVI with zero-contrast strategy using the self-expandable Evolut R/Pro THV in patients with chronic kidney disease. The non-contrast approach included three major steps. (1) Feasibility of the transfemoral approach and the entry site determined by non-contrast multidetector computed tomography (MDCT) and an aortoiliac CO_2_ angiography in the pre-procedural work-up. (2) Detection of high-risk anatomy for annulus rupture, severe paravalvular leak (PVL), or coronary occlusion based on non-contrast MDCT. Patients with high-risk anatomy were excluded. THV sizing was based on (i) sinus of Valsalva (SOV) mean diameter assessed by non-contrast MDCT and (ii) annulus perimeter (AP) assessed by 3D-TEE and non-contrast magnetic resonance imaging (MRI) by two methods, 3D whole-heart and cine SSFP. (3) TAVI procedures were performed under general anesthesia and guided by TEE and fluoroscopy, using the optimal projection defined by the non-contrast MDCT.

Patient safety was ensured by important actions (safety checkpoints) during this pilot phase of the study (Figure 1). Initially, although the procedural planning and the choice of the device size were based on non-contrast imaging modalities, patients underwent standard ECG-gated thoracic and aortoiliac contrast MDCT, which was blinded to the TAVI operator, but not other experienced members of the heart team. The contrast MDCT was used to (1) assure the feasibility of the TF TAVI (safety checkpoint 1); (2) confirm that high-risk anatomies for annulus rupture and coronary occlusion have been excluded (safety checkpoint 2); and (3) confirm that the size of the THV chosen was not undersized for the aortic annulus.

Safety checkpoints were also included during the TAVI procedures using the “zero-contrast” technique. As soon as the position of the device was considered optimal by the operators, right before the final THV release, a standard contrast aortogram was obtained to ensure that an acceptable position of the THV was achieved (safety checkpoint 3). Furthermore, at the end of the procedure, the access site was evaluated primarily with a non-contrast CO2 angiography, but a contrast iliofemoral angiography was also performed to exclude any vascular complication (safety checkpoint 4). At any moment, the primary operator was allowed to use contrast media to identify or treat any complication related to the procedure.

### Procedure/intervention description

The TAVI procedures were performed under general anesthesia, since TEE was chosen to guide the THV deployment and assess the result of the procedure, in special with regard to THV positioning and the presence of paravalvular leak (PVL). All procedures were performed using the TF approach, and arterial access was guided by ultrasound, fluoroscopy, and CO_2_ angiography. The jugular venous access was used to place a temporary pacemaker in all cases. To streamline the interventional treatment of possible vascular complications, a 0.018” guidewire in the common femoral artery was inserted from the contralateral side in selected patients with borderline access. The decision of whether to use pre-dilatation was left to the discretion of the operator. The implant of the THV was guided by TEE and fluoroscopy, using the cusp-overlap projection defined by the non-contrast MDCT. A pigtail catheter positioned at the non-coronary cusp and calcium markers at the level of the annulus were used as references for the deployment, to achieve a THV position of 3–5 mm below the native annulus. The option to recapture and reposition the valve was used to achieve the intended position. After THV release, post-dilatation was performed in case of significant PVL that was identified exclusively by the TEE and hemodynamic assessment. At the end of the procedure, a final contrast aortogram was performed to guarantee that a good result was achieved and for the assessment of the depth of THV and residual PVL. Vascular access site closure was achieved with two ProGlide devices (Abbott Vascular, USA). At the end of the intervention, the hemostasis of the entry site was confirmed by ultrasound and CO_2_ angiography.

### Statistical method

The sample size was subjectively defined as an exploratory series of cases to serve as a reference for a larger future study. Statistical analysis was performed using SPSS software for statistical computing. Continuous variables were presented as mean ± SD or median (interquartile range). Categorical variables were presented as percentages. Kolmogorov–Smirnov test was used to test the normality of the variable. The *χ*^2^ test was used to determine the association between two categorical variables. The Bland–Altman test was used to assess the level of agreement between imaging methods and Pearson to evaluate the strength and direction of the linear relationship. The Wilcoxon signed-rank test was used to verify eventual differences between imaging methods. Receiver operating characteristic (ROC) analysis was used to identify optimal cutoff points of the relationship between SOV and annulus and better understand oversizing cases. A probability value <0.05 was considered significant.

## Results

### Patient characteristics

Demographic and baseline characteristics are shown in Table 1. From December 2020 to December 2021, a total of 29 patients with severe AS were enrolled, and four patients were excluded due to unsuitable femoral access, subaortic membrane, high-risk anatomy with severe left ventricular outflow tract calcification, and presence of severe mitral prosthesis regurgitation, respectively. There was no need for interference in case selection based on information from the contrast MDCT (safety checkpoint). The final population of the study comprised 25 patients, with a mean age of 79 years, 13 (52%) were male, in NYHA functional class III/IV in 72%, and the mean creatinine clearance was 49 ± 7 ml/min. The mean STS and EuroScore II risk of mortality were 3.0% ± 1.5% and 3.7% ± 3.2%, respectively. Regarding the anatomical characteristics, 20% had bicuspid morphology, 32% had some degree of LVOT calcification, and the mean aortic valve calcium score was 3.728 (Agatston).

**Table 1:** Clinical and echocardiographic characteristics of the study population.

Clinical variables	All patients (*n* = 25)
Male	13 (52%)
Age mean	79.9 ± 6.1
BMI mean	26.0 ± 3.7
NYHA—functional class III/IV	18 (72%)
STS—PROM	3.0 ± 1.5
EuroScore II	3.7 ± 3.2
eGFR	49 (44–56)
EFT	1.1 ± 1.3
Prior hospitalization 3 months	11 (44%)
Hypertension	20 (80%)
Diabetes	9 (36%)
Chronic lung disease	12 (48%)
Prior stroke	2 (8%)
Coronary artery disease	14 (56%)
Prior CABG	2 (8%)
Prior PCI	6 (24%)
Bicuspid valve	5 (20%)
LVOT calcification	8 (32%)
Aortic valve calcium score (Agatston)	3,728 ± 1,400
Echocardiographic variables	
LVEF	59 ± 11
LVEF < 35%	3 (12%)
Aortic valve orifice area (cm²)	0.67 ± 16.0
Aortic valve orifice area index (cm²/m^2^)	0.38 ± 0.08
Mean aortic valve gradient (mmHg)	54.0 ± 19.7
PASP (mmHg)	34.0 ± 9.5
Moderate/severe mitral regurgitation	1 (4%)
Baseline rhythm	
Sinus rhythm	20 (80%)
Atrial fibrillation/flutter	3 (12%)
Pacemaker	2 (8%)
Conduction disturbances	
LBBB	2 (8%)
RBBB	2 (8%)
First-degree block	4 (16%)

Values are *n* (%), mean (±SD), or median [IQR]; BMI, body mass index; NYHA, New York Heart Association; STS-PROM, Society of Thoracic Surgeons Predicted Risk of Mortality; eGFR, estimated glomerular filtration; EFT, essential frailty toolset; CABG, coronary artery bypass graft coronary; PCI, percutaneous coronary intervention; LVOT, left ventricular outflow tract; LVEF, left ventricular ejection fraction; PSAP, pulmonary artery systolic pressure; LBBB, left bundle branch block; RBBB, right bundle branch block.

The preoperative echocardiographic evaluation revealed a mean left ventricular ejection fraction (LVEF) of 59.0% ± 11.2%, with three (12%) patients with an LVEF < 35%, a mean aortic valve orifice area index of 0.38 ± 0.08 cm^2^/m^2^, and a mean transaortic valve gradient of 54.0 ± 19.7 mmHg (Table 1).

### Procedural details

Procedural details are shown in Table 2. A self-expandable Evolut R and Pro THV were implanted in 80% and 20% of patients, respectively, with high rates of pre-dilation (68%) and post-dilatation (75%). The cusp overlap technique (implantation plane chosen by the non-contrast MDCT) was used for the THV implantation in all cases, and the mean implantation depth was 6.0 ± 2.3 mm when measured by angiography and 4.5 ± 1.8 mm when evaluated by TEE. The mean contrast volume used for the safety checkpoints and final aortogram was 45 ± 35 ml. A new pacemaker was implanted in four patients (16% overall and 17.4% of the patients at risk). The median length of in-hospital stay was 5 (3–9) days.

**Table 2:** Procedure characteristics.

Clinical variables	All patients (*n* = 25)
THV size	
23 mm	1 (4%)
26 mm	6 (24%)
29 mm	13 (52%)
34 mm	5 (20%)
Type *n* (%)	
Evolut R	20 (80%)
Evolut Pro	5 (20%)
Depth within the LVOT	
Fluoroscopic	6.0 ± 2.3
TEE	4.5 ± 1.8
Recapture	16 (64%)
Number of recapture	1 (0–3)
Pre-dilation	17 (68%)
Post-dilation	19 (75%)
Contrast (ml)	45.0 ± 35.2
Rapid pacing	22 (88%)

Values are *n* (%), mean (±SD), or median [IQR]; TEE, transesophageal echocardiogram; LVOT, left ventricular outflow tract.

### Primary safety endpoint

The primary early safety endpoint was achieved in 92%. There was no mortality or stroke in this pilot phase. One (4%) patient developed major bleeding due to a vascular complication that occurred in the contralateral access site and 1 (4%) presented calcium embolization to the coronary artery, with no need for intervention. A total of 6 (24%) patients presented with AKI, 5 (20%) stage I and 1 (4%) stage II, with no need for dialysis (Table 3).

**Table 3:** Endpoints.

Combined primary safety endpoint at 30 days (VARC-2)	92%
Device success (VARC-2)	92%
All-cause mortality	0
Major stroke (disabling and non-disabling)	0
Life-threatening bleeding	0
Coronary artery obstruction requiring intervention	0
Major bleeding	1 (4%)
Major vascular complication	1 (4%)
Repeat procedure for valve-related dysfunction.	0
Acute kidney injury (AKI)	6 (24%)
AKI stage 1	5 (20%)
AKI stages 2–3	1 (4%)
Need for dialysis	0
Myocardium infarction	1 (4%)
New LBBB post-procedure	7/19 (36%)
New pacemaker	4/23 (17%)

Values are *n* (%) or mean (±SD); VARC-2, valve academic research consortium−2; AKI, acute kidney injury; LBBB, left bundle branch block.

### Secondary safety endpoint

**Figure 2:**
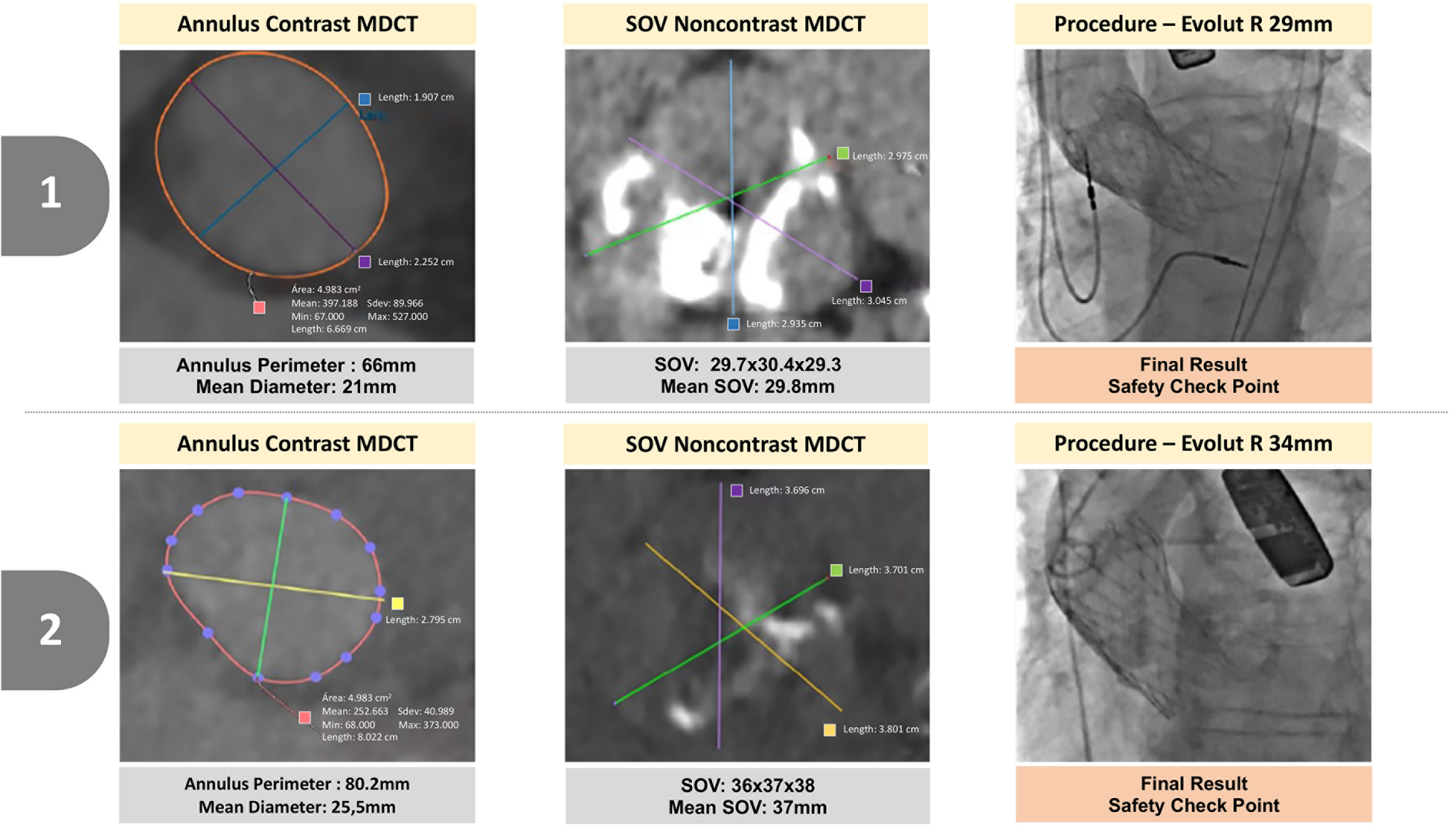
Two illustrative cases in which oversized prostheses were implanted. Patients more prone to receive an oversized THV. (1) Patients with a large SOV in relation to the annulus size (relation SOV/annulus >1.37) and (2) patients with a borderline annular size, between two sizes of the Evolut prosthesis.

Device success was achieved in 92% (Table 3). All valves were chosen and released as intended and none needed repositioning, post-dilatation, or any modification in the procedure after the contrast use at the safety checkpoints. One THV embolization requiring second valve implantation ensued in a patient with a severely calcified aortic valve and sigmoid septum, probably because of the lack of pre-dilatation, even though a perfect release position was confirmed by the safety aortogram. With regard to THV sizing, an oversized THV (one size larger) was implanted in 9 (36%) cases as compared to the gold-standard annulus sizing by contrast MDCT. Patients more prone to receive an oversized THV were those with a borderline annular size, between two sizes of the Evolut prosthesis, and also those with a large SOV in relation to the annulus size (relation SOV/annulus >1.37; AUC: 0.819; *p* = 0.009) (Figure 2). In these nine oversized cases, no adverse events were observed, although a numerically higher rate of PPM was observed (22% vs. 12%; *p* = 0.5). No cases of undersized THV were detected; therefore, in 64% of the cases, the size was chosen in agreement with the instructions for the use of the THV. At 30 days, all patients were alive, and in NYHA class I/II, and two patients (8%) with heavily calcified valves presented with moderate PVL (Evolut R implanted). Echocardiographic findings revealed a mean aortic valve gradient of 7 ± 3.8 mmHg and an effective orifice area of 1.90 ± 0.34 cm^2^ (Table 4).

**Table 4:** Follow up at 30 days.

All-cause death	0
Stroke/TIA	0
Myocardium infarction	0
Hospital readmission	0
NYHA—functional class *n*. (%)	
I	21 (84%)
II	3 (12%)
III	1 (4%)
SAPT	17 (68%)
DAPT	5 (20%)
OAC	3 (12%)
Creatinine	1.1 ± 0.2
Hemoglobin	11.6 ± 1.5
Mean aortic valve gradient (mmHg)	7.0 ± 3.8
Effective orifice area (cm^2^)	1.9 ± 0.3
Aortic regurgitation	
None/trivial	15 (60%)
Mild	8 (32%)
Moderate	2 (8%)
Severe	0

Values are *n* (%) and mean (±SD); ICU, intensive care unit; TIA, transient ischemic attack; NYHA, new york heart association; SAPT, single antiplatelet therapy; DAPT, dual antiplatelet therapy, OAC, oral anticoagulation.

### Comparison between imaging methods

No significant differences were detected between the annulus perimeter measured by the gold-standard contrast MDCT and by 3D-TEE and cardiac 3D whole-heart MRI. With regard to the SOV, the size measured by contrast MDCT was smaller than by non-contrast MDCT, larger than by 3D-TEE, and equivalent to the sizing by MRI. Coronary ostium height by contrast MDCT was larger than that by non-contrast MDCT and 3D-TEE. There was no difference in the size of the femoral vessels when evaluated by contrast MDCT or CO_2_ angiography (Table 5). Also, we observed a good correlation of the cusp overlap implantation plane obtained by the non-contrast and contrast MDCT. The relationship of each measurement between non-contrast assessments and contrast MDCT using correlation and Bland–Altman plots is shown in Supplementary Figure S1.

**Table 5:** Comparison between imaging methods.

Variables (*n* = 25)	Mean	Std. deviation	Minimum	Maximum	25th percentile	50th percentile (median)	75th percentile	*p* *
**Annulus perimeter contrast MDCT**	73.4	6.4	61.5	88.4	68.4	70.2	78.4	
Annulus perimeter 3D-TEE	73.4	8.2	54.0	89.0	68.7	69.0	80.3	0.276
Annulus perimeter Cine-MRI	75.3	4.7	65.2	85.0	72.8	75.0	78.6	0.194
Annulus perimeter WH-MRI	75.3	6.7	65.0	88.0	69.3	74.0	80.7	0.178
**Annulus area contrast MDCT**	416.8	73.6	290	602	361.5	379.0	471.5	
Annulus area 3D-TEE	422.0	89.0	210	82	361.0	390.0	490.0	0.635
Annulus area Cine-MRI	442.0	58.5	320	540	410.0	431.5	497.2	0.123
Annulus area WH-MRI	430.2	74.1	320	570	365.0	430.0	499.5	0.728
**SOV contrast MDCT**	31.3	3.7	25.2	37.0	28.7	30.2	35.3	
SOV non-contrast MDCT	32.0	4.0	25.9	38.8	28.9	32.1	36.1	0.006
SOV 3D-TEE	30.1	3.6	23.6	36.0	27.0	29.5	33.4	0.004
SOV Cine-MRI	31.4	3.4	26.2	36.7	28.3	31.2	34.7	0.338
SOV WH-MRI	31.9	3.3	26.8	36.8	29.0	31.9	35.4	0.394
**LM height contrast MDCT**	14.4	2.3	10.7	19.6	12.5	14.4	16.3	
LM height non-contrast MDCT	13.2	1.7	9.5	16.7	12.4	12.9	14.1	0.006
LM height 3D-TEE	12.7	2.7	9.0	19.0	11.0	13.0	14.0	0.016
**RC height contrast MDCT**	16.1	3.6	11.0	24.7	13.4	15.0	19.3	
RC height non-contrast MDCT	14.7	3.7	9.5	24.0	11.9	14.1	17.2	0.003
RC height 3D-TEE	13.6	2.6	9.0	20.0	12.0	13.0	15.0	0.001
**RFA diameter contrast MDCT**	7.0	1.1	5	10.0	6	7.1	7.9	
RFA diameter CO_2_ Angiography	6.7	0.8	5.2	9.1	6.0	6.5	7.2	0.089

TEE, transesophageal echocardiogram; MDCT, multidetector computed tomography; MRI, magnetic resonance imaging; WH, whole heart; SOV, sinus of Valsalva; LM, left main; RC, right coronary; RFA, right femoral artery.

*p-values represent comparison against the gold-standard contrast MDCT.

## Discussion

This is the first prospective study to evaluate a full non-contrast approach for transfemoral TAVI among patients with severe symptomatic AS and CKD. In this pilot study, using a self-expandable THV, we have shown that this new non-contrast strategy for procedural planning and THV implantation is feasible and safe, with similar results as compared to previous studies using the standard technique with contrast (12, 13). In the present study, device success and the combined primary safety endpoint (at 30 days) both achieved 92%. The study protocol intentionally included contrast use at important checkpoints, to guarantee the safety of the new strategy in this initial series. Also, we choose the Evolut R/Pro valve because it has some specific characteristics (small profile, self-expandable, recapturable, and repositionable) favoring the implementation of a zero-contrast TAVR technique. In addition, a self-expandable device allows for a less precise sizing than a balloon expandable THV, which might be key when using non-contrast imaging modalities for procedural planning.

The aortic valve and LVOT calcification can be easily evaluated by non-contrast CT, which is key for procedural planning. Also, since non-contrast methods such as TEE and MDCT underestimate coronary height when compared to contrast MDCT, we can assume that these methods can be safely used to identify those at higher risk for coronary obstruction.

The size of the Evolut (EV) R/Pro THV was defined based on the sinus of Valsalva mean diameter as assessed by the non-contrast MDCT and, in borderline situations, the annulus perimeter assessed by 3D TEE and by non-contrast MRI was also used to define THV size. We have demonstrated that the size of the SOV can be easily evaluated by the non-contrast MDCT and that this measurement is usually 1–2 mm larger than the one by contrast MDCT, probably because the non-contrast method includes the thickness of the aortic wall into its measurement. With regard to annulus perimeter, several studies showed that measurements of the aortic annulus by 3D-TEE yield comparable results with those obtained by MDCT, which is in perfect agreement with our findings (14). Our study shows, in addition, that non-contrast 3D whole-heart MRI is another imaging modality that can be used to evaluate annulus size. In the present study, an oversized THV was chosen in one-third of the patients, with no adverse events occurring in all of them, clearly demonstrating that oversizing a self-expandable THV for the annulus is safe, provided that there is enough space to accommodate the device in the SOV, to avoid coronary occlusion and eventually sinus sequestration. However, we do recognize that oversizing a THV might increase the need for PPM and this issue deserves further investigation. On the other hand, undersizing a self-expandable THV could be much more problematic, due to the increased risk of paravalvular leak and THV embolization, but it never occurred in this initial series using non-contrast imaging modalities to size the device.

The implantation procedure was fully guided by TEE and fluoroscopy. Using this technique, we were able to achieve a good implantation position in all cases, which was confirmed by one of the predefined safety checkpoints with contrast media. Even the case presenting with THV embolization had a good position before the full release of the device. One limitation of the present technique is the need for TEE guidance during the procedure, to guarantee that the position and deepness of the THV are adequate before full release. Another potential drawback is that, without contrast, there might be a tendency of the operator to release the THV deeper into the LVOT, which might expose these patients to a higher risk of PPM (15). However, in our series, the rate of PPM of 17.4% was very similar to the 17% observed in the Evolut Low Risk trial, using the standard implantation technique with contrast (12).

CO_2_ angiography used for planning and during the TAVI procedure is another novelty of the present zero-contrast TAVI technique, and we have shown that it can provide information regarding sizing, obstructions, and tortuosity and also detect access site complications (16).

Finally, although the main advantage of the present technique is not to expose patients to the nephrotoxicity of the contrast media, we acknowledge that the reduction of contrast volume may not always lead to a decreased risk of AKI. Other factors, such as hypotension, bleeding, and inflammation, among others, might also play an important role in the occurrence of AKI after TAVI.

## Conclusion

Based on the present results, we believe that this new “zero-contrast” technique for procedural planning and self-expandable THV implantation might become the preferable strategy for a significant population of CKD patients undergoing TAVR. Of course, the results of this pilot study deserve confirmation in a larger series to be implemented in clinical practice. Therefore, the next phase of the study using no contrast at all (without safety checkpoints) in a larger series of CKD patients is warranted.

## Data Availability

The raw data supporting the conclusions of this article will be made available by the authors, without undue reservation.

## References

[1] Najjar M Salna M George I. Acute kidney injury after aortic valve replacement: incidence, risk factors and outcomes. Expert Rev Cardiovasc Ther. (2015) 13(3):301–16. 10.1586/14779072.2015.1002467 25592763

[2] Hibino M Yoon SH Dallan LAP Pelletier MP Rushing GD Filby SJ Feasibility and safety of exclusive noncontrast computed tomography for planning of transcatheter aortic valve implantation with self-expandable valves. Am J Cardiol. (2023) 190:122–4. 10.1016/j.amjcard.2022.12.018 36623398

[3] Kappetein AP Head SJ Généreux P Piazza N van Mieghem NM Blackstone EH Updated standardized endpoint definitions for transcatheter aortic valve implantation: the valve academic research consortium-2 consensus document. J Thorac Cardiovasc Surg. (2013) 145(1):6–23. 10.1016/j.jtcvs.2012.09.002 23084102

[4] Gargiulo G Sannino A Capodanno D Perrino C Capranzano P Barbanti M Impact of postoperative acute kidney injury on clinical outcomes after transcatheter aortic valve implantation: a meta-analysis of 5,971 patients: AKI impacts on TAVI outcomes. Cathet Cardiovasc Intervent. (2015) 86(3):518–27. 10.1002/ccd.25867 25641565

[5] Elhmidi Y Bleiziffer S Deutsch MA Krane M Mazzitelli D Lange R Acute kidney injury after transcatheter aortic valve implantation: incidence, predictors and impact on mortality. Arch Cardiovasc Dis. (2014) 107(2):133–9. 10.1016/j.acvd.2014.01.002 24556191

[6] Codner P Orvin K Assali A Sharony R Vaknin-Assa H Shapira Y Long-term outcomes for patients with severe symptomatic aortic stenosis treated with transcatheter aortic valve implantation. Am J Cardiol. (2015) 116(9):1391–8. 10.1016/j.amjcard.2015.08.004 26342515

[7] Rosa VEE Campos CM Bacelar A Abizaid AAC Mangione JA Lemos PA Performance of prediction models for contrast-induced acute kidney injury after transcutaneous aortic valve replacement. Cardiorenal Med. (2021) 11(4):166–73. 10.1159/000517058 34261063

[8] Nunes Filho ACB Katz M Campos CM Carvalho LA Siqueira DA Tumelero RT Impact of acute kidney injury on short- and long-term outcomes after transcatheter aortic valve implantation. Rev Esp Cardiol (Engl Ed). (2019) 72(1):21–9. 10.1016/j.rec.2017.11.024 29358043

[9] de Brito FS Carvalho LA Sarmento-Leite R Mangione JA Lemos P Siciliano A Outcomes and predictors of mortality after transcatheter aortic valve implantation: results of the Brazilian registry: hTAVI outcomes and predictors of mortality. Cathet Cardiovasc Interv. (2015) 85(5):E153–62. 10.1002/ccd.25778 25510532

[10] Gebauer K Diller GP Kaleschke G Kerckhoff G Malyar N Meyborg M The risk of acute kidney injury and its impact on 30-day and long-term mortality after transcatheter aortic valve implantation. Int J Nephrol. (2012) 2012:1–8. 10.1155/2012/483748 PMC3541560 23365748

[11] Coca SG Yusuf B Shlipak MG Garg AX Parikh CR. Long-term risk of mortality and other adverse outcomes after acute kidney injury: a systematic review and meta-analysis. Am J Kidney Dis. (2009) 53(6):961–73. 10.1053/j.ajkd.2008.11.034 19346042 PMC2726041

[12] Popma JJ Deeb GM Yakubov SJ Mumtaz M Gada H O’Hair D Transcatheter aortic-valve replacement with a self-expanding valve in low-risk patients. N Engl J Med. (2019) 380(18):1706–15. 10.1056/NEJMoa1816885 30883053

[13] Reardon MJ Van Mieghem NM Popma JJ Kleiman NS Søndergaard L Mumtaz M Surgical or transcatheter aortic-valve replacement in intermediate-risk patients. N Engl J Med. (2017) 376(14):1321–31. 10.1056/NEJMoa1700456 28304219

[14] Hahn RT Nicoara A Kapadia S Svensson L Martin R. Echocardiographic imaging for transcatheter aortic valve replacement. J Am Soc Echocardiogr. (2018) 31(4):405–33. 10.1016/j.echo.2017.10.022 29275985

[15] Pascual I Hernández-Vaquero D Alperi A Almendarez M Avanzas P Kalavrouziotis D Permanent pacemaker reduction using cusp-overlapping projection in TAVR. JACC Cardiovasc Interv. (2022) 15(2):150–61. 10.1016/j.jcin.2021.10.002 35057985

[16] Cho KJ. Carbon dioxide angiography: scientific principles and practice. Vacc Specialist Iint. (2015) 31(3):67–80. 10.5758/vsi.2015.31.3.67 PMC4603680 26509137

