# Supplementary material for: Non-contrast transcatheter aortic valve implantation for patients with aortic stenosis and chronic kidney disease: a pilot study
Source: Front Cardiovasc Med. 2023 Jun 14;10:1175600. doi: 10.3389/fcvm.2023.1175600 (PMC10305775; doi:10.3389/fcvm.2023.1175600)
Supplement: Supplementary file 1 [file Datasheet1.docx]

| **Table S1.**  Size of the Evolut R/Pro THV based on the sinus of Valsalva (SOV) mean diameter as assessed by the noncontrast MDCT. |
| --- |
| **Size of the Evolut R/Pro SOV me diameter**  23mm. <27 mm |
| 26mm 27-28.9 mm  29mm. 29-30.9 mm  34mm ≥31mm |

Supplementary Material

THV = transcatheter heart valve; SOV = sinus of valsalva; MDCT = multidetector computed tomography

| **Table S2.** Criteria Inclusion and Exclusion |
| --- |
| Inclusion criteria  1. Severe Aortic Stenosis  2. Evaluated and accepted for TAVI by the heart team  3. CKD stage ≥3a (glomerular filtration rate < 60mL/min/1,73m2)  Exclusion criteria  1. Any condition considered a contraindication for placement of a bioprosthetic valve (eg, patient is indicated for mechanical prosthetic valve).  2. A known hypersensitivity or contraindication to any of the following that cannot be adequately pre-medicated:  a. aspirin, clopidogrel or heparin (HIT/HITTS)  b. nitinol (titanium or nickel)  c. contrast media (pilot phase)  3. Blood dyscrasias as defined: leukopenia (white blood cell count <1000mm3), thrombocytopenia (platelet count < 50,000 cells/mm3), history of bleeding diathesis or coagulopathy, or hypercoagulable states.  4. Ongoing infection, including active endocarditis.  5. Cardiogenic shock manifested by low cardiac output, vasopressor dependence, or mechanical hemodynamic support.  6. Recent (within 2 months of Heart Team assessment) cerebrovascular accident or transient ischemic attack.  7. Gastrointestinal bleeding that would preclude anticoagulation.  8. Patient refuses a blood transfusion.  9. Severe dementia (resulting in either inability to provide informed consent for the trial/procedure, prevents independent lifestyle outside of a chronic care facility, or will fundamentally complicate rehabilitation from the procedure or compliance with follow-up visits).  10. Estimated life expectancy of less than 12 months due to associated non-cardiac comorbid conditions.  11. Other medical, social, or psychological conditions that, in the opinion of the investigator, precludes the patient from appropriate consent or adherence to the protocol required follow-up exams.  12. Pre-existing prosthetic heart valve at aortic position.  13. Presence of severe mitral stenosis or regurgitation.  14. Presence of hypertrophic obstructive cardiomyopathy with left ventricular outflow gradient.  15. Clinical indication for coronary angiography or percutaneous coronary intervention in the period including the 30 days before and continuing for the 30 days after the TAVI procedure. The use of contrast media for these procedures can be a confounding factor for the analysis of the endpoint of the study.  16. Contraindication for the transfemoral approach based on the evaluation by noncontrast MDCT and by aortoiliac CO2 angiography (anatomical screening).  17. High-risk for annulus rupture or severe PVL (heavy LVOT calcification) or coronary occlusion (coronary ostium height <10 mm with narrow SOV: < 1mm larger than the size of the THV) defined by imaging methods. |

TAVI = Transcatheter aortic valve implantation; CKD = chronic kidney disease; HIT = heparin induced thrombocytopenia; HITTS = heparin induced thrombocytopenia with thrombosis; MDCT = multidetector computed tomography; CO2 = carbon dioxide; PVL = paravalvular leak; LVOT = left ventricular outflow tract; SOV = Sinus of Valsalva; THV = transcatheter heart valve.

**Figure S1**: Relationship between non-contrast assessments and contrast MDCT with Pearson correlation and Bland-Altman analyses

**A:** Pearson correlation between annulus perimeter by contrast MDCT and 3D TEE, MRI and MRIWH.

**B:** Bland-Altman analyses between annulus perimeter by contrast MDCT and 3D TEE, MRI and MRIWH.

**C:** Pearson correlation between annulus area by contrast MDCT and 3D TEE, MRI and MRIWH.

**D:** Bland-Altman analyses between annulus perimeter by contrast MDCT and 3D TEE, MRI and MRIWH.

**E:** Pearson correlation between SOV by contrast MDCT and noncontrast MDCT, 3D TEE, MRI and MRIWH.

**F:** Bland-Altman analyses between SOV perimeter by contrast MDCT and 3D TEE, MRI and MRIWH.

**G:** Pearson correlation between LM height by contrast MDCT and noncontrast MDCT and 3D TEE.

**H:** Bland-Altman analyses between LM height by noncontrast MDCT and 3D TEE.

**I:** Pearson correlation between RC height by contrast MDCT and noncontrast MDCT and 3D TEE.

**J:** Bland-Altman analyses between RC height by noncontrast MDCT and 3D TEE.

**K:** Pearson correlation between RFA diameter by contrast MDCT and CO2 angiography.

**L:** Pearson correlation between projection deployment (cusp-overlap) by contrast MDCT and noncontrast MDCT

MDCT= Multidetector computer tomography; TEE = transesophageal echocardiogram; MRI = magnetic resonance imaging; WH = whole heart; RC = Right coronary; SOV = Sinus of Valsalva; LM = Left main; RFA = Right femoral artery.
