# Supplementary figures and images for: Non-contrast transcatheter aortic valve implantation for patients with aortic stenosis and chronic kidney disease: a pilot study
Source: Front Cardiovasc Med. 2023 Jun 14;10:1175600. doi: 10.3389/fcvm.2023.1175600 (PMC10305775; doi:10.3389/fcvm.2023.1175600)

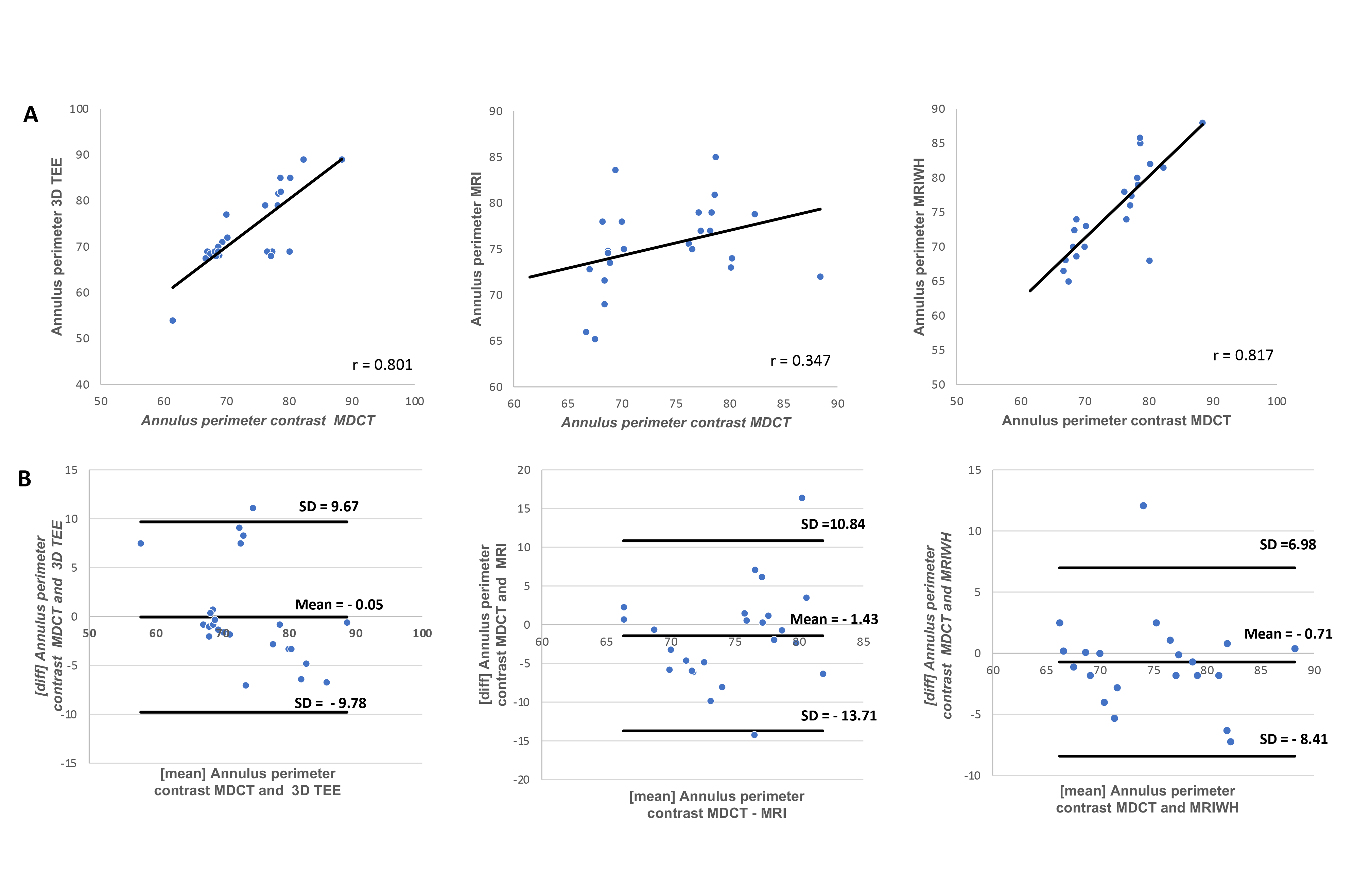

Supplement: Supplementary file 2 [file Image1.tif]

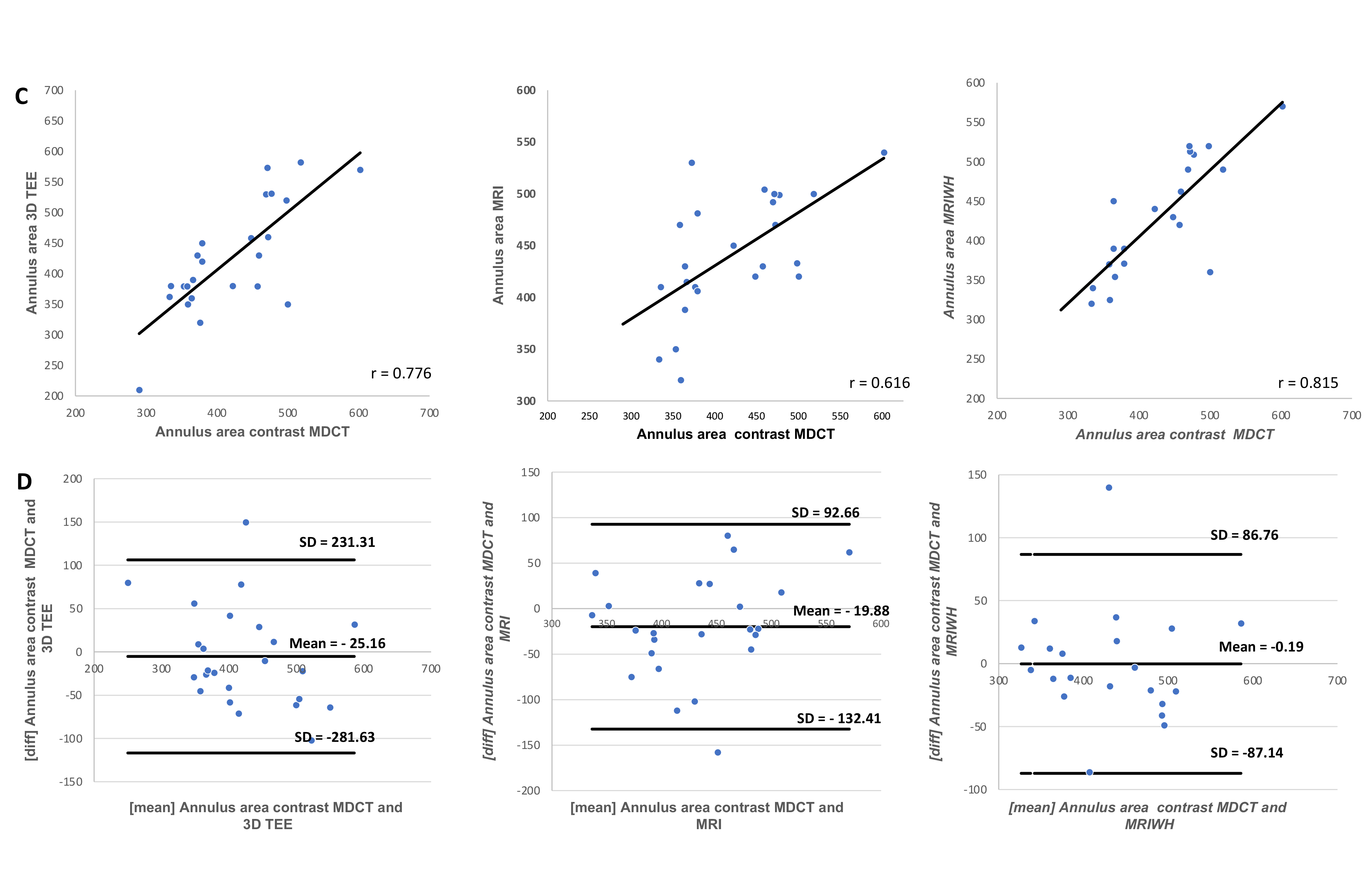

Supplement: Supplementary file 3 [file Image2.tif]

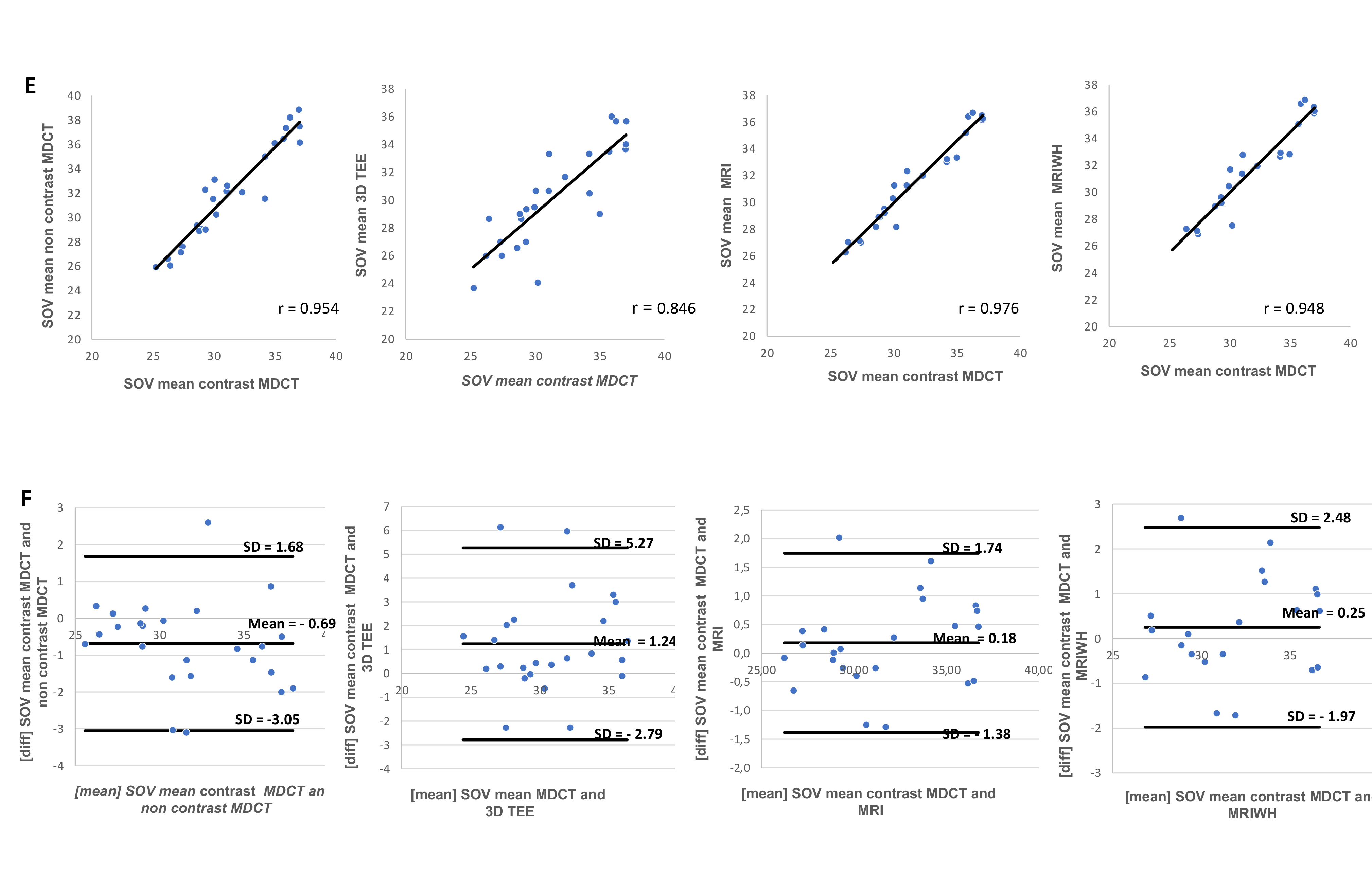

Supplement: Supplementary file 4 [file Image3.tif]

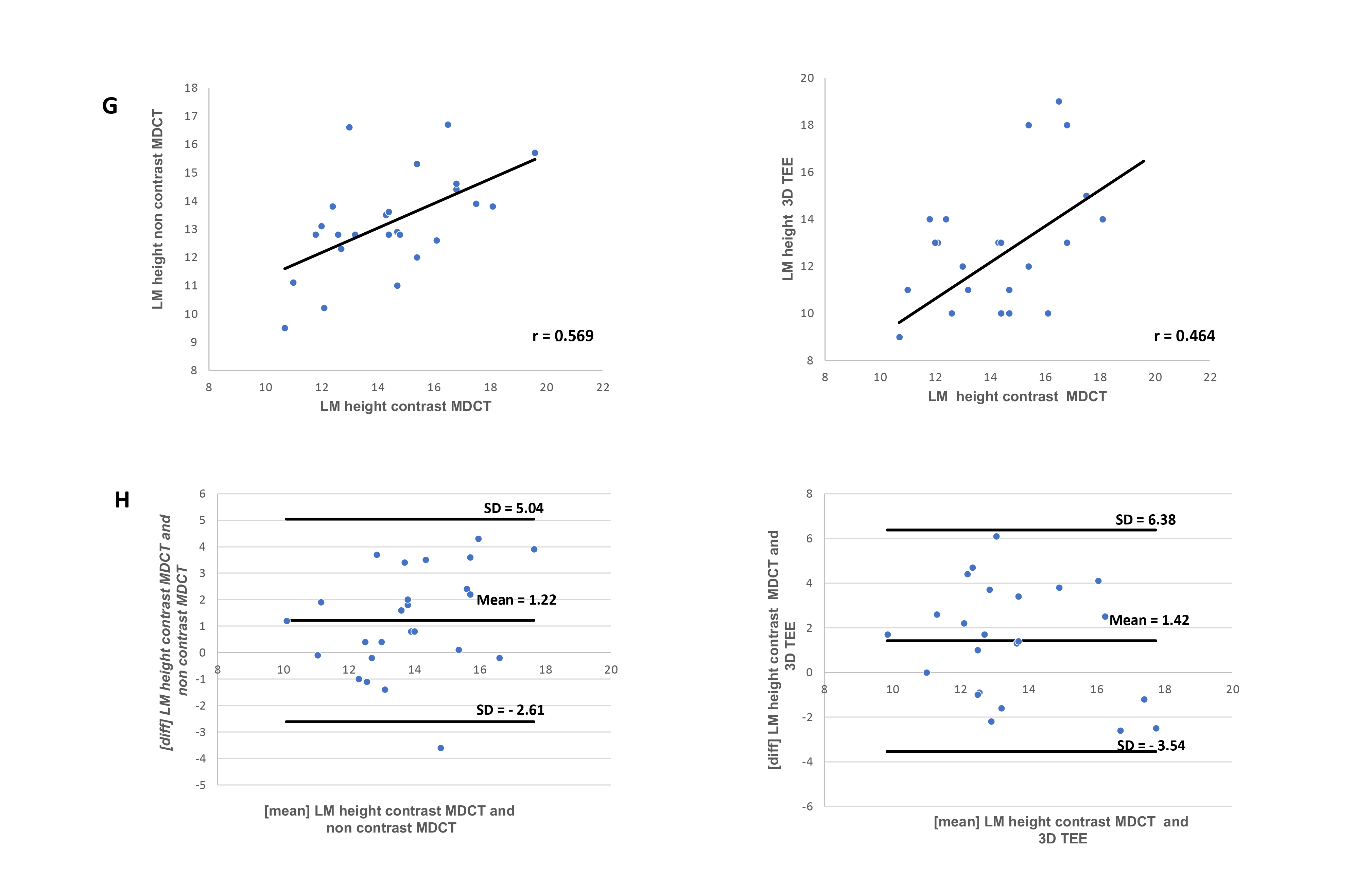

Supplement: Supplementary file 5 [file Image4.tif]

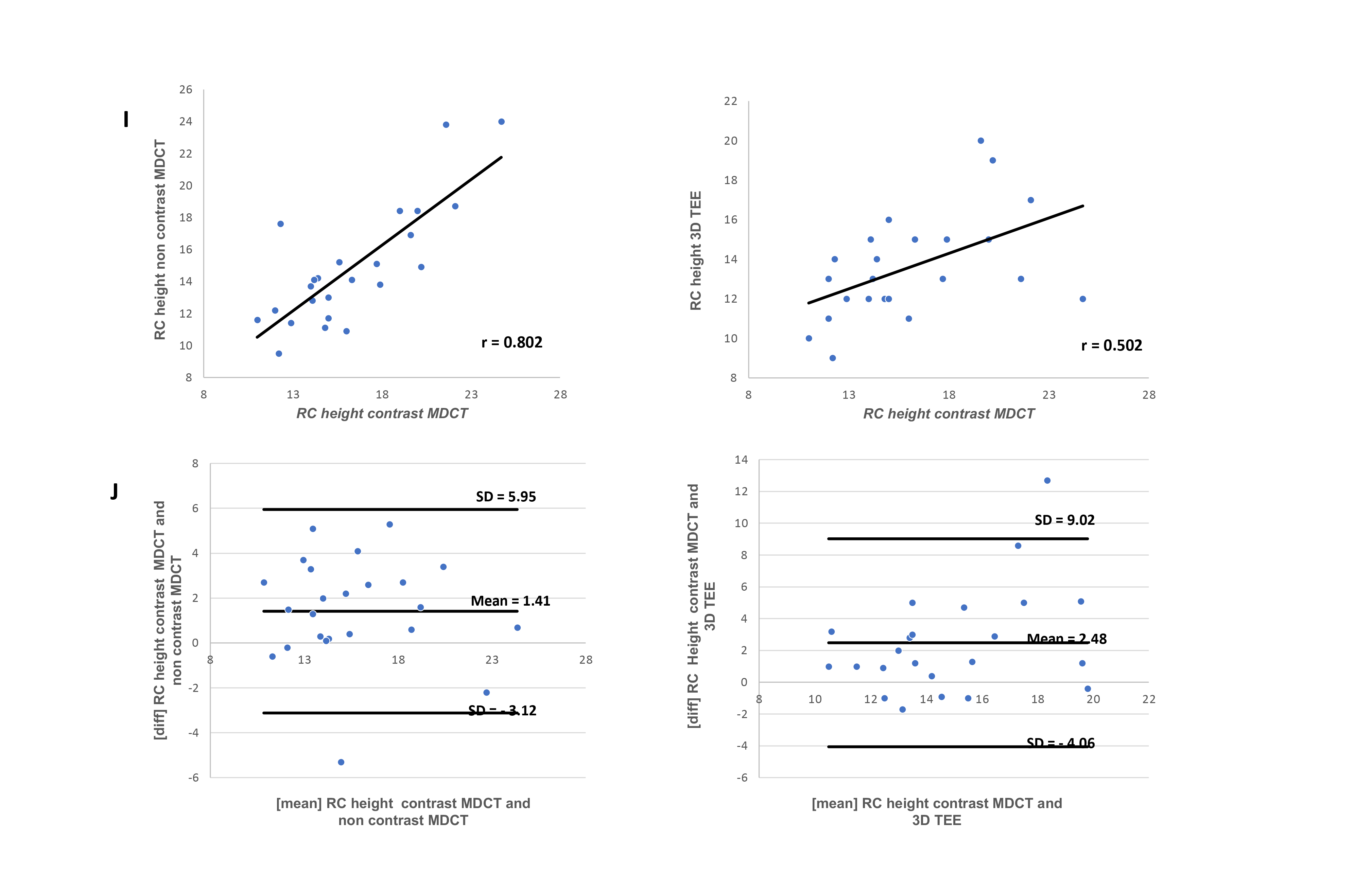

Supplement: Supplementary file 6 [file Image5.tif]

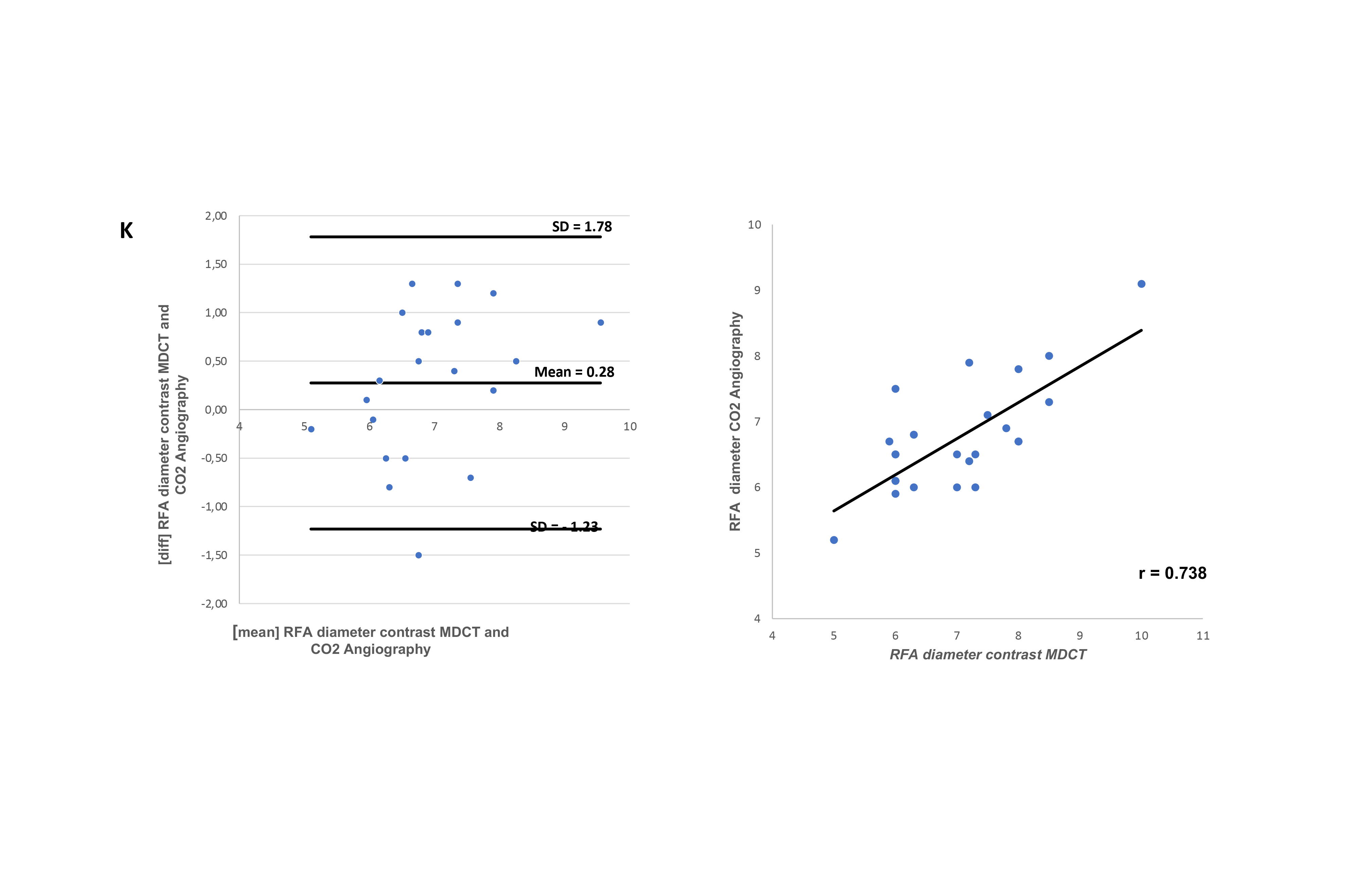

Supplement: Supplementary file 7 [file Image6.tif]

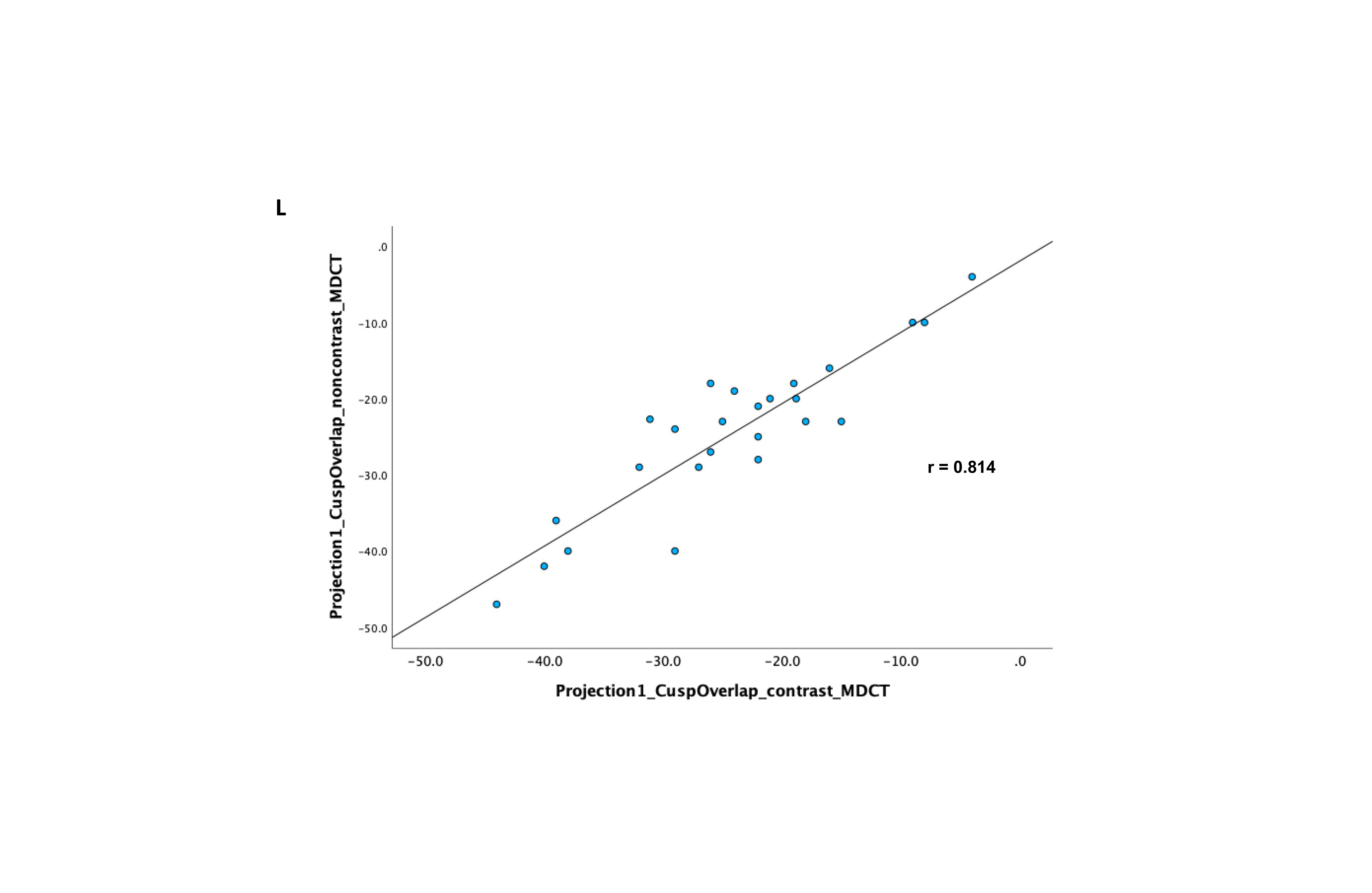

Supplement: Supplementary file 8 [file Image7.tif]
